# Soil bacterial community characteristics and influencing factors in different types of farmland shelterbelts in the Alaer reclamation area

**DOI:** 10.3389/fpls.2024.1488089

**Published:** 2024-10-28

**Authors:** Cuiping Tian, Xue Wu, Bota Bahethan, Xianyao Yang, Qianqian Yang, Xiantao Wang

**Affiliations:** ^1^ College of Ecology and Environment, Xinjiang University, Urumqi, China; ^2^ Key Laboratory of Oasis Ecology, Ministry of Education, Xinjiang University, Urumqi, China; ^3^ Xinjiang Jinghe Observation and Research Station of Temperate Desert Ecosystem, Ministry of Education, Jinghe, China; ^4^ Technology Innovation Center for Ecological Monitoring and Restoration of Desert-Oasis, Ministry of Natural Resources (MNR), Urumqi, China

**Keywords:** Alaer reclamation area, farmland shelterbelts, soil properties, soil microorganisms, correlation network analysis

## Abstract

To investigate the effects of various types of farmland shelterbelts on soil quality and soil bacterial community diversity, this study focused on soil samples from four different shelterbelt types in the Alaer reclamation area, including *Populus euphratica* Oliv.- *Populus tomentosa* Carrière (PP), *Elaeagnus angustifolia* L.- *Populus euphratica* Oliv. (EP), *Populus alba* var. *pyramidalis* Bunge (P), and *Salix babylonica* L. (S). We analyzed their physical, chemical, biological properties as well as the differences in bacterial community structure, and explored the influencing factors on soil microbial community characteristics through microbial correlation network analysis. The results showed that: (1) There were significant differences in soil properties among the four types of farmland shelterbelts (*p* < 0.05), with P soils exhibiting the highest levels of organic matter, total nitrogen, and total phosphorus contents. (2) The Alpha diversity indices of soil bacteria showed significant differences among the four types of farmland shelterbelts (*p* < 0.05), with the P soils displayed the highest Chao1 and Shannon indices. (3) There were differences in the composition and abundance of dominant soil bacterial communities among different farmland shelterbelts, notably, the abundances of Verrucomicrobia, Acidobacteria, and Planctomycetes were significantly higher in P soils compared to the other three types. (4) The complexity of the correlation network between microbial species and environmental factors was highest in EP soils, soil microbial biomass nitrogen and available phosphorus were the main influencing factors. These findings indicated that different types of farmland shelterbelts had significant impacts on soil properties and soil bacterial communities. Soil bacterial communities were regulated by soil properties, their changes reflected a combined effect of soil characteristics and tree species.

## 1 Introduction

The farmland shelterbelt system (FSS) is an essential component of agroforestry systems and a key ecological barrier against adverse environmental conditions (Krichen et al., 2017; Wang et al., 2020). This system is widely applied in ecologically fragile areas facing challenges of wind erosion and soil desertification, such as the northern regions of China, including Northwest and Northeast China (Bao et al., 2012; Cerdà et al., 2022). By designing and constructing different types of shelterbelt networks around farmland, FSS aims to resist sand and wind disasters, improve soil properties and quality, increase soil microbial diversity, and promote ecosystem health (Chen et al., 2017; Luca et al., 2018; Cao et al., 2021). In the context of increasingly severe global environmental issues such as climate change, land degradation, and biodiversity loss, the importance of FSS is gradually being recognized worldwide (Haddaway et al., 2018; Fang, 2021). Especially in enhancing agricultural sustainability and ecological restoration, FSS is regarded as an effective ecological engineering measure (Kong et al., 2022).

The surface soil of forest land is an important habitat for microorganisms, many of which are beneficial. The diversity and activity of forest soil microorganisms play a crucial role in the decomposition of organic matter, the release of nutrients, the promotion of matter and energy cycling, and the balance and maintenance of the health and stability of forest soil ecosystems (Breulmann et al., 2012; Edwards et al., 2015; Hacquard and Schadt, 2015; Fang et al., 2016). Previous studies have shown that the abundance of Actinobacteria varies the most among different types of farmland shelterbelts (Zhang J. et al., 2019), while the Proteobacteria and Acidobacteria are most affected by afforestation (Li et al., 2019; Deng et al., 2019a). Actinobacteria can produce extracellular polysaccharides and other substances, improving soil aeration and water retention capacity. Proteobacteria promote nitrogen cycling in the soil, and Acidobacteria are involved in the decomposition of organic matter, all of which have significant effects on soil improvement and nutrient cycling. Studying the characteristics of these microorganisms and their interactions with environmental factors will help improve our understanding of the dynamics of forest soil ecosystems and provide a scientific basis for ecological restoration and management.

Soil microorganisms are extremely sensitive to environmental changes and are influenced by various factors, including soil properties, climate, vegetation, and root activity. Farmland shelterbelts increase the biomass of surface litter and root activity, enhancing the decomposition rate of organic matter and nutrient content in the soil, thereby positively regulating soil microorganisms. Studies have shown that the establishment of farmland shelterbelts significantly increases soil microbial biomass, metabolism, and diversity (Jan et al., 2014; Qiu et al., 2017; Liu et al., 2019). Therefore, it is crucial to explore the impact of soil properties on the structure and diversity of microbial communities. High soil organic matter content promotes processes such as microbial organic matter degradation, nitrogen fixation, and mineral transformation, leading to changes in soil microbial community structure. Soil pH and nitrogen are also key factors determining soil microbial diversity and composition. Previous research results have shown a significant positive correlation between pH and microbial diversity and richness (Bao et al., 2012; Liu et al., 2014). Total nitrogen and alkali-hydrolyzable nitrogen can also directly affect the relative abundance of dominant bacterial phyla (Deng et al., 2019b). The environment regulates the distribution of soil microorganisms, while the characteristics of soil microbial community respond to soil ecological processes and vegetation changes (Rodrigues et al., 2015). However, few studies have comprehensively analyzed and evaluated the combined effects of different types of FSS vegetation changes and understory soil properties on soil microbial community characteristics. Additionally, reports using 16S rDNA high-throughput sequencing technology to study FSS soil microorganisms are relatively scarce.

The Alaer reclamation area is adjacent to the Taklamakan Desert, where frequent wind and sand activities occur. The study indicates that the soil organic matter content in the Aral reclamation area is relatively low, classifying it as lightly salinized soil (Cai et al., 2013). The spatial variation of soil water and salt is significantly influenced by soil texture and topography (Xu and Sun, 2023). Through reclamation and cultivation management, it is possible to effectively improve the degree of soil salinization and significantly increase the microbial biomass in the soil (Wang et al., 2021; Guo et al., 2023). The establishment of Farmland shelterbelts could effectively prevent wind and sand disasters, maintaining high-quality and stable agricultural development and benefiting the construction of high-standard farmland. On this background, it is necessary to study the influence of different types of FSS on soil bacterial community characteristics, which could reveal the ecological protection function of shelterbelts from multiple dimensions. *Populus euphratica* Oliv., *Populus tomentosa* Carrière, *Populus alba* var. *pyramidalis* Bunge, *Elaeagnus angustifolia* L., Salix babylonica L. are commonly used tree species for constructing farmland shelterbelts in the Alaer reclamation area. This study measured and analyzed the soil properties and microbial community structure under different types of farmland shelterbelts during the growing season. In this context, the following questions are proposed: (1) How do soil properties vary among different types of farmland shelterbelts? (2) How do soil bacterial community compositions respond to different types of farmland shelterbelts? (3) What are the influencing factors of soil bacterial community characteristics in different types of farmland shelterbelts? The results of this study will provide a theoretical basis and technical support for the sustainable development of farmland shelterbelt soil in this region, ensuring the long-term stable effectiveness of protective benefits.

## 2 Materials and methods

### 2.1 Overview of the study area

The Alaer reclamation area is located in the heart of the Eurasian continent, in the northern part of the Tarim Basin, at the confluence of the Aksu River, Yarkand River, and Hotan River, in the upper reaches of the Tarim River. Its geographical coordinates are between 40°22′ - 40°57′ N and 80°30′ - 81°58′ E, stretching 281 km from east to west and 180 km from north to south. The region experiences a warm temperate extreme continental arid desert climate. It receives 2800-3000 hours of sunshine annually, has a frost-free period of 200-220 days, an average annual precipitation of about 75 mm, an annual evaporation rate of 1200-1500 mm, and an average annual temperature of approximately 10°C. Natural disasters in the area include wind and sand, droughts, cold waves, and floods, with wind and sand being the most prevalent natural disaster. The primary soil types identified in the area include brown desert soil and saline-alkaline soil.

**Figure 1:**
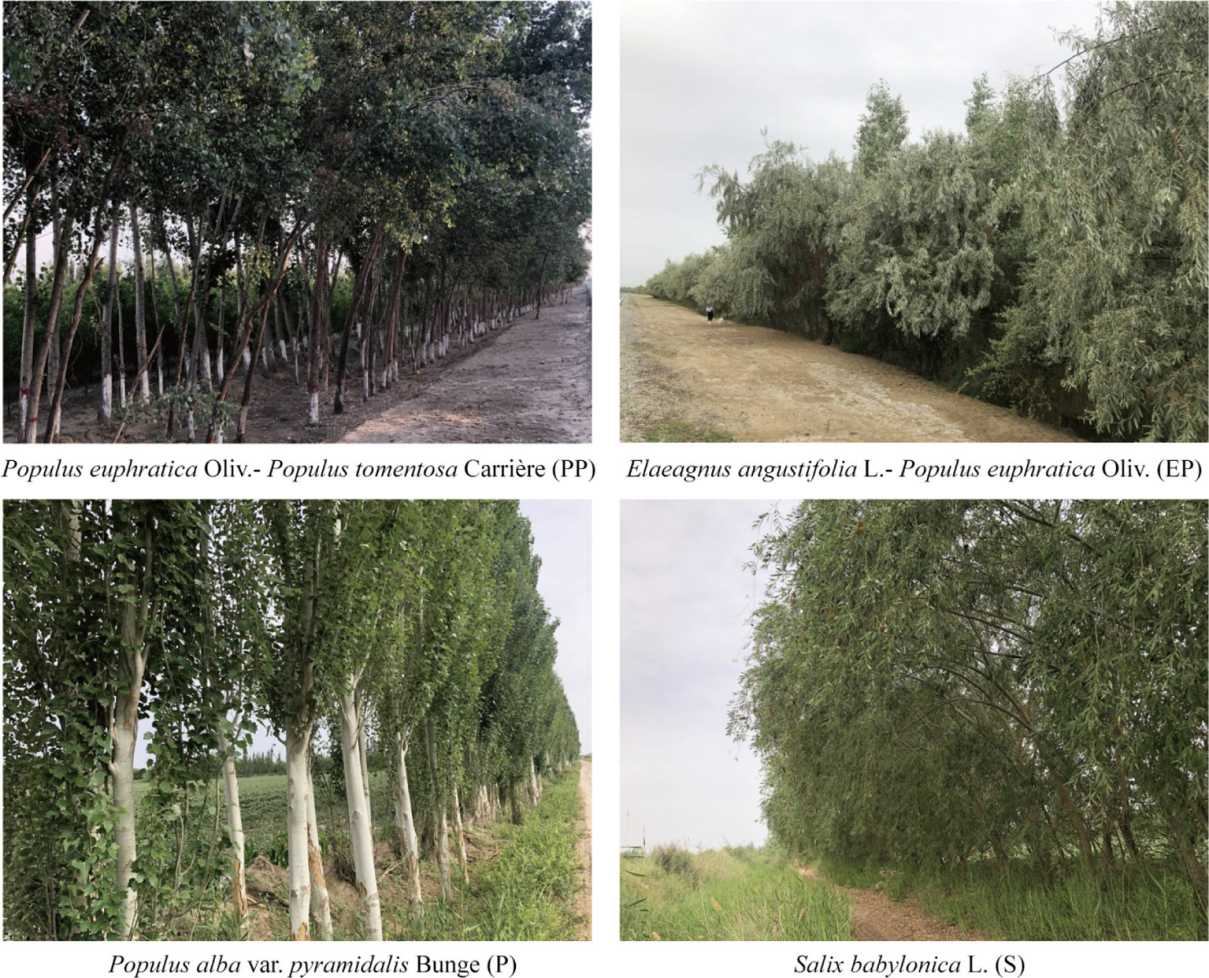
Field photos of different types of farmland shelterbelts.

Following a field survey, four types of farmland shelterbelts were selected as research subjects: *Populus euphratica* Oliv.- *Populus tomentosa* Carrière farmland shelterbelt (PP), *Elaeagnus angustifolia* L.- *Populus euphratica* Oliv. farmland shelterbelt (EP), *Populus alba* var. *pyramidalis* Bunge farmland shelterbelt (P), and *Salix babylonica* L. farmland shelterbelt (S) (**Figure 1**).

### 2.2 Soil sample collection

In June 2021, field surveys were conducted in around the city of Alaer. In each type of farmland shelterbelt, three sample plots of 2m × 5m were established in areas with similar site conditions, 12 sample plots in total. The trees in each plot were measured for height and diameter at breast height to determine the average tree height and diameter (**Table 1**). Based on measurements, one healthy tree without disease or pest damage was selected as the standard tree for each plot.

**Table 1:** The structure of different types of farmland shelterbelts.

	Configuration	Average height of trees/m	Diameter at breast height/cm
PP	*Populus euphratica* Oliv. /*Populus tomentosa* Carrière	10.5/16	12.5/21
EP	*Elaeagnus angustifolia* L. /*Populus euphratica* Oliv.	6.5/10.5	15/12.5
P	*Populus alba* var. *pyramidalis* Bunge	22.5	26
S	*Salix babylonica* L.	14.5	13.5

Under the canopy of standard trees, near the base of roots, surface litter and debris were removed. Using a sterile shovel, soil samples were collected at depth of 0-20 cm, with three repetitions. After mixing the three repetitions thoroughly, the soil was sieved through a 2 mm sieve and divided into three portions. One portion was placed in an aluminum box for soil moisture content determination, one portion was placed in a sterile cryotube, taken back to the laboratory, and stored at -80°C for soil bacterial and microbial biomass determination. The remaining portion was air-dried for pH, electrical conductivity, soil organic carbon, available phosphorus, alkali-hydrolyzable nitrogen, total nitrogen, and total phosphorus determination.

### 2.3 Soil property analysis

Soil properties were determined according to *Soil Agricultural Chemistry Analysis* (Bao, 2000), pH was measured using the potentiometric method (soil to water ratio 1:5); electrical conductivity (EC) was measured using the conductivity method (soil to water ratio 1:5); soil water content (SWC) was determined using the drying method; soil organic carbon (SOC) was determined using the potassium dichromate volumetric method; available phosphorus (AP) was determined using the sodium bicarbonate extraction method; alkali-hydrolyzable nitrogen (AN) was determined using the alkaline diffusion method; total nitrogen (TN) was determined using the Nessler’s reagent spectrophotometric method; and total phosphorus (TP) was determined using the molybdenum antimony anti-colorimetric method.

Soil microbial biomass was determined according to *Soil Microbial Biomass Determination Methods and Applications* (Wu et al., 2006). Microbial biomass carbon (MBC), microbial biomass nitrogen (MBN), and microbial biomass phosphorus (MBP) were all determined using the chloroform fumigation extraction method.

### 2.4 Soil DNA extraction and high-throughput sequencing

The TGuide S96 magnetic bead method soil genomic DNA extraction kit (Model: DP812, Tiangen Biotech (Beijing) Co., Ltd.) was used to extract total DNA from soil microorganisms in different types of farmland shelterbelts according to the kit’s manual. The nucleic acid concentration was measured using a microplate reader (Manufacturer: Gene Company Limited, Model: Synergy HTX), and amplification was performed based on the measurement results. For bacterial communities, universal bacterial primers 27F (5’-AGRGTTTGATYNTGGCTCAG-3’) and 1492R (5’-TASGGHTACCTTGTTASGACTT-3’) were used to amplify the full-length region of the bacterial 16S rDNA gene sequence. The PCR products were electrophoresed on 1.8% agarose gel (Manufacturer: Beijing Biotech-Xin Technology Co., Ltd.) to check for integrity. The PCR reaction conditions were as follows: 95°C for 2 min; 25 cycles of denaturation at 98°C for 10 s, annealing at 55°C for 30 s, and extension at 72°C for 1 min 30 s; and a final extension at 72°C for 2 min.

For the constructed libraries, the PacBio SMRTbell Template Prep Kit was used for damage repair, end repair, and adapter ligation of the mixed products. The reaction process was conducted on a PCR instrument, and the final library was recovered using AMpure PB magnetic beads. The PacBio Binding kit was used to bind the library with primers and polymerase before sequencing on the Sequel II sequencer, sequencing and sequence alignment work were undertaken by Beijing Biomarker Technologies Co., Ltd.

### 2.5 Bioinformatics analysis and data processing

The raw subreads obtained from sequencing were corrected to obtain Circular Consensus Sequencing (CCS) sequences using SMRT Link, version 8.0. The lima software (v1.7.0) was then used to identify CCS sequences of different samples through barcode sequences and to remove chimeras, resulting in Effective CCS sequences. The Effective CCS sequences were clustered into Operational Taxonomic Units (OTUs) at a similarity level of 97% using Usearch v10.0. Using the SILVA reference database, taxonomic annotation of the feature sequences was conducted with a Naïve Bayes classifier in combination with alignment methods. This approach allows for the statistical analysis of community composition at various levels (phylum, class, order, family, genus, species) for each sample.

Data organization and analysis were performed using Excel 2019 and IBM SPSS Statistics 26 software. One-way analysis of variance (One-way ANOVA) using the Waller-Duncan test was employed, and results were presented as mean ± standard error. Origin 2023 software was used for plotting. QIIME 2 software was used to calculate Alpha diversity indices (ACE, Chao1, Simpson, and Shannon). Principal component analysis (PCA) was conducted using R software. The Biomarker Microbial Diversity Analysis Platform (www.biocloud.net) was used for correlation analysis between soil microbial species and environmental factors.

## 3 Results

### 3.1 Differences in soil properties among different types of farmland shelterbelts

One-way analysis of variance showed significant differences in soil TP, AP, AN, pH, EC, SWC, MBC, MBP, and MBN among the four types of farmland shelterbelts (**Table 2**). Among them, P had the highest SOC, TP, TN, AP, AN, SWC, MBC, MBP, and MBN among the four types of farmland shelterbelts, with TP, AP, AN, SWC, MBC, MBP, and MBN significantly higher than the other three types of farmland shelterbelts (*p* < 0.05). The EC of P was the lowest, showing significant differences compared to the other types of farmland shelterbelts (*p* < 0.05). The soils of all four types of farmland shelterbelts were alkaline (pH > 8.3), with S having the highest pH, significantly higher than the other three types of farmland shelterbelts (*p* < 0.05).

**Table 2:** Soil properties of different types of farmland shelterbelts.

	PP	EP	P	S
SOC	4.55 ± 0.26	4.92 ± 0.59	6.03 ± 1.56	3.82 ± 0.40
TP	0.61 ± 0.02**AB**	0.53 ± 0.02**B**	0.65 ± 0.01**A**	0.60 ± 0.04**AB**
TN	0.28 ± 0.01	0.37 ± 0.04	0.46 ± 0.11	0.25 ± 0.02
AP	4.61 ± 1.02**C**	2.62 ± 0.42**C**	32.74 ± 0.54**A**	7.10 ± 0.67**B**
AN	25.45 ± 1.46**B**	33.03 ± 2.41**AB**	46.58 ± 9.36**A**	23.56 ± 0.86**B**
pH	8.73 ± 0.05**AB**	8.49 ± 0.07**BC**	8.30 ± 0.10**C**	8.92 ± 0.13**A**
EC	1538.33 ± 150.58**C**	3926.67 ± 135.44**A**	883.00 ± 83.19**D**	2592.67 ± 197.99**B**
SWC	5.84 ± 0.61**B**	2.42 ± 0.20**C**	12.05 ± 1.02**A**	3.76 ± 0.08**BC**
MBC	45.52 ± 4.55**C**	38.47 ± 2.13**C**	115.41 ± 0.46**A**	73.78 ± 0.81**B**
MBP	4.23 ± 0.38**BC**	6.60 ± 0.89**AB**	7.59 ± 0.89**A**	3.60 ± 0.41**C**
MBN	3.36 ± 0.33**B**	4.25 ± 0.88**B**	11.47 ± 0.36**A**	4.35 ± 1.01**B**

Data in the table are means ± standard error; different capital letters in the same row indicate significant differences at the *p* = 0.05 level.

### 3.2 Soil bacterial community characteristics and differences among different types of farmland shelterbelts

**Figure 2:**
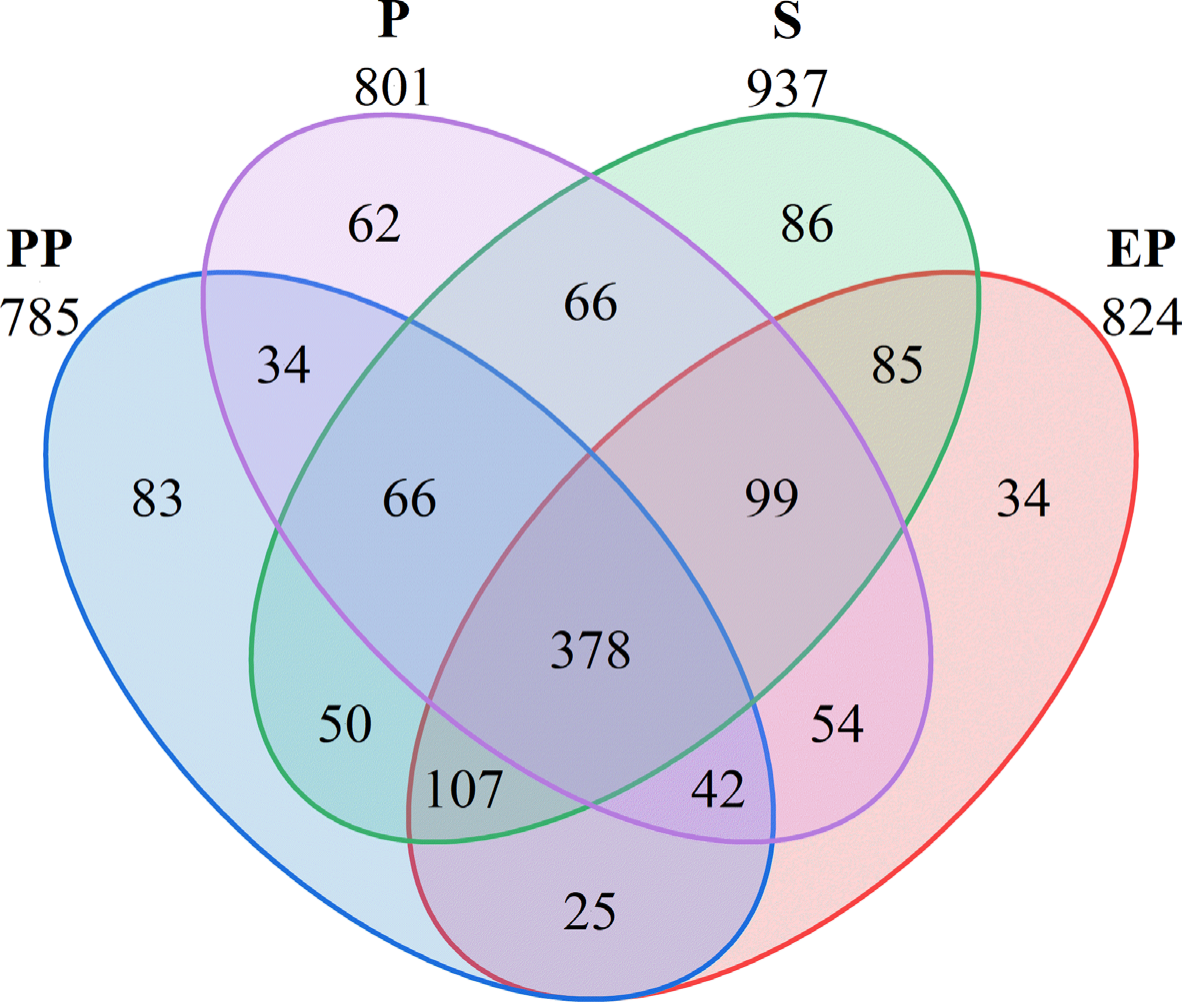
Venn diagram of different types of farmland shelterbelts based on OTUs.

A total of 1271 OTUs were detected in the soil bacterial communities of the four types of farmland shelterbelts, with 378 OTU shared among all samples, accounting for 29.74%. The number of unique OTU was highest in S (86), followed by PP (83), P (62), and EP (34). S had the highest total OTU count as well as the highest number of unique OTUs, while EP had the second-highest total OTU count but the lowest number of unique OTU. P had a higher total OTU count than PP but a lower number of unique OUT (**Figure 2**).

The coverage indices of all samples were above 0.97. The Chao1 and Shannon indices of EP were significantly lower than those of P (*p* < 0.05), but showed no significant differences compared to PP and S. However, the ACE index of EP was significantly lower than the other three types of farmland shelterbelts. There were no significant differences in the Simpson index among the four types of farmland shelterbelts (*p* > 0.05) (**Table 3**).

**Table 3:** Alpha diversity analysis of different types of farmland shelterbelts soils.

	PP	EP	P	S
ACE	589.88 ± 48.84**A**	370.76 ± 6.05**B**	660.57 ± 49.86**A**	611.92 ± 83.39**A**
Chao1	555.38 ± 85.32**AB**	372.20 ± 1.18**B**	655.75 ± 43.64**A**	580.90 ± 89.79**AB**
Simpson	0.96 ± 0.02	0.96 ± 0.00	0.99 ± 0.00	0.97 ± 0.01
Shannon	6.47 ± 0.57**AB**	5.96 ± 0.01**B**	7.91 ± 0.02**A**	6.72 ± 0.82**AB**
Coverage	0.97 ± 0.00**B**	0.99 ± 0.00**A**	0.97 ± 0.01**AB**	0.98 ± 0.00**AB**

Data in the table are means ± standard error; different capital letters in the same row indicate significant differences at the 0.05 level.

Based on the taxonomic analysis of soil genomic DNA sequences, a total of 23 bacterial phyla and 433 bacterial genera were detected in all soil samples. Specifically, 15 phyla and 204 genera were detected in PP, 16 phyla and 185 genera in EP, 17 phyla and 259 genera in P, and 15 phyla and 201 genera in S.

**Figure 3:**
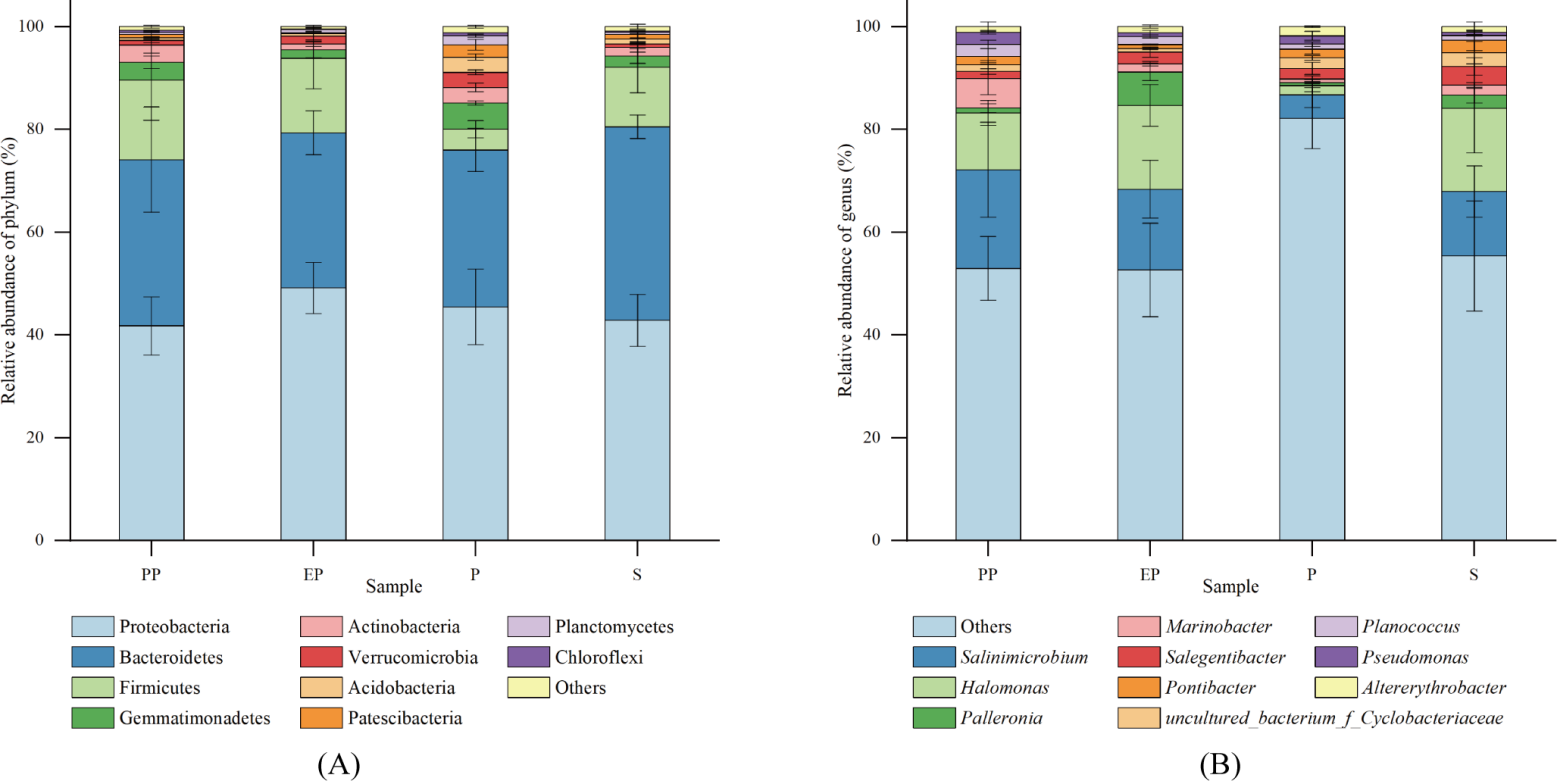
Relative abundance of soil bacterial communities at phylum **(A)** and genus **(B)** levels in different types of farmland shelterbelts.

The top 10 phyla in relative abundance were selected for each type of shelterbelt soil, and a relative abundance bar stack chart was generated (**Figure 3A**). Proteobacteria was the dominant phylum in all samples, accounting for 30.80% to 58.72% of the total sequences, followed by Bacteroidetes (11.96% - 44.09%), Firmicutes (0.88% - 29.93%), Gemmatimonadetes (0.11% - 5.90%), Actinobacteria (0.58% - 6.45%), Verrucomicrobia (0.13% - 3.82%), Acidobacteria (0.02% to 3.63%), Patescibacteria (0.08% - 4.11%), Planctomycetes (0.12% - 2.18%), and Chloroflexi (0.01% - 0.61%). Proteobacteria, Bacteroidetes, and Firmicutes were the common dominant phyla in the four types of farmland shelterbelts. Bacterial phyla with a relative abundance of more than 1% in P were more abundant than the other three types of farmland shelterbelt.

At the genus level, the proportion of dominant genera was relatively low (**Figure 3B**), with *Salinimicrobium* (0.09% - 35.96%) and *Halomonas* (0.23% - 31.55%) being the dominant in all four types of farmland shelterbelts. Then it was followed by *Palleronia* (0.08% - 9.05%), *Marinobacter* (0.04% - 11.79%), *Salegentibacter* (0.07% - 5.49%), *Uncultured_bacterium_f_Cyclobacteriaceae* (0.38% - 7.04%), *Pontibacter* (0.03% - 6.39%), *Planococcus* (0.27% - 4.00%), *Pseudomonas* (0.10% - 3.36%), and *Altererythrobacter* (0.05% - 2.92%).

The soil bacteria in the four types of farmland shelterbelts showed differences in the composition and abundance of some dominant groups. The abundance of the Verrucomicrobia, Acidobacteria, and Planctomycetes in P was significantly higher than in the other three types of farmland shelterbelts, while the abundance of *Palleronia* in EP was significantly higher. There were no significant differences in the other dominant community at the phylum and genus levels (*p*> 0.05) (**Table 4**).

**Table 4:** Differences in soil bacterial community composition at phylum and genus levels in different types of farmland shelterbelts.

		PP	EP	P	S
	Proteobacteria	41.77 ± 5.67	49.15 ± 4.97	45.48 ± 7.34	42.84 ± 5.06
	Bacteroidetes	32.35 ± 10.24	30.20 ± 4.27	30.51 ± 4.19	37.65 ± 2.28
	Firmicutes	15.47 ± 7.79	14.5 ± 5.95	4.05 ± 1.66	11.61 ± 4.98
	Gemmatimonadetes	3.50 ± 1.21	1.64 ± 1.53	5.12 ± 0.41	2.19 ± 1.43
	Actinobacteria	3.32 ± 1.60	1.16 ± 0.51	3.01 ± 0.82	1.69 ± 0.94
Phylum	**Verrucomicrobia**	**0.92 ± 0.46B**	**1.48 ± 0.71AB**	**2.93 ± 0.48A**	**0.65 ± 0.17B**
	**Acidobacteria**	**0.51 ± 0.24B**	**0.51 ± 0.50B**	**2.98 ± 0.62A**	**0.94 ± 0.90AB**
	Patescibacteria	0.62 ± 0.35	0.15 ± 0.11	2.40 ± 1.03	0.91 ± 0.69
	**Planctomycetes**	**0.46 ± 0.08B**	**0.66 ± 0.42B**	**1.75 ± 0.28A**	**0.44 ± 0.30B**
	Chloroflexi	0.37 ± 0.13	0.10 ± 0.09	0.53 ± 0.05	0.23 ± 0.17
	Others	0.72 ± 0.31	0.45 ± 0.23	1.24 ± 0.28	0.85 ± 0.45
	Others	52.96 ± 6.20	52.65 ± 9.09	82.17 ± 5.95	55.38 ± 10.74
	*Salinimicrobium*	19.21 ± 9.24	15.73 ± 5.60	4.57 ± 2.46	12.53 ± 5.01
	*Halomonas*	11.05 ± 2.42	16.27 ± 4.06	1.77 ± 1.17	16.2 ± 8.66
	***Palleronia***	**0.94 ± 0.85B**	**6.55 ± 1.67A**	**0.63 ± 0.23B**	**2.57 ± 1.49AB**
	*Marinobacter*	5.71 ± 3.11	1.60 ± 0.44	0.65 ± 0.63	1.90 ± 0.56
Genus	*Salegentibacter*	1.44 ± 0.55	2.27 ± 0.97	2.10 ± 1.20	3.64 ± 1.70
	*uncultured_bacterium_f_Cyclobacteriaceae*	1.31 ± 0.79	0.64 ± 0.18	2.04 ± 0.47	2.70 ± 2.17
	*Pontibacter*	1.57 ± 1.51	0.82 ± 0.11	1.72 ± 1.00	2.42 ± 1.99
	*Planococcus*	2.35 ± 0.83	1.59 ± 0.32	0.95 ± 0.43	0.96 ± 0.17
	*Pseudomonas*	2.39 ± 0.36	0.71 ± 0.45	1.62 ± 0.87	0.56 ± 0.36
	*Altererythrobacter*	1.07 ± 0.92	1.18 ± 0.31	1.79 ± 0.17	1.13 ± 0.93

Data in the table are means ± standard error; different capital letters in the same row indicate significant differences at the 0.05 level.

Bolded numbers highlight results that are significantly different between groups.

### 3.3 Factors affecting soil bacterial community characteristics

**Figure 4:**
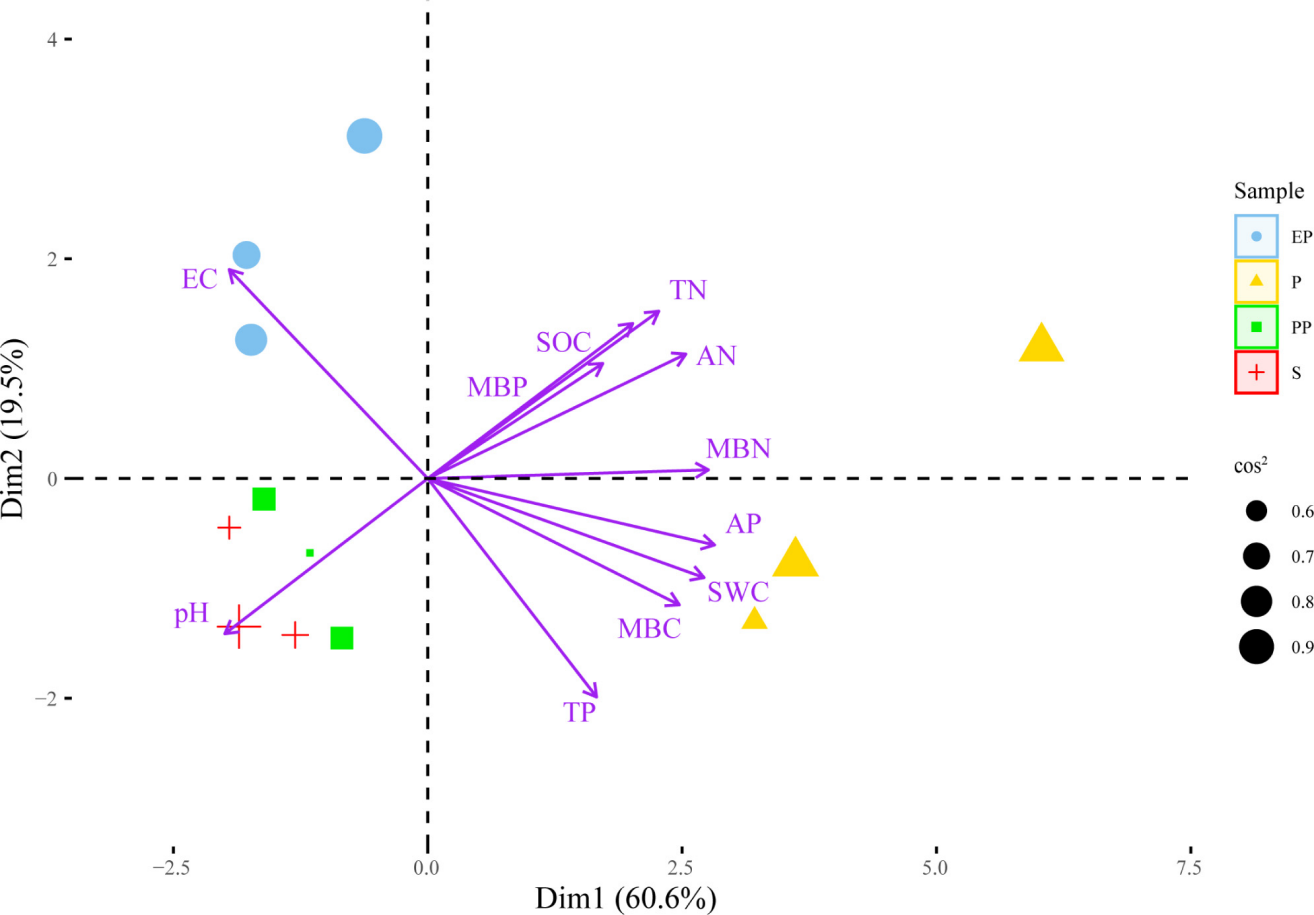
Principal component analysis of soil environment and bacterial community structure in different types of farmland shelterbelts.

In the PCA plot (**Figure 4**), the angle between the arrows and the distance projected onto the PC axis indicate that MBN, AP, AN, SWC, TN, MBC, SOC, TP, and MBP are positively correlated with PC1, while EC and pH are negatively correlated with PC1. The lengths of arrow of pH, EC, TN were longer, suggesting that they had greater contributions to PC1 and PC2.

According to the principle of eigenvalues > 1, a total of 3 principal components were extracted (**Table 5**), with contribution rates of 60.57%, 19.54%, and 10.89% respectively, cumulatively contributing to 91.00%. Soil indicators with factor loadings absolute value ≥ 0.75 were grouped into one component. TN, AP, AN, SWC, MBC, and MBN had high loadings on the first principal component, with values of 0.768, 0.954, 0.858, 0.919, 0.837, and 0.934 respectively.

**Table 5:** Load matrix of principal components.

	Dim.1	Dim.2	Dim.3
SOC	0.681	0.478	0.525
TP	0.561	-0.673	0.253
TN	0.768	0.515	0.376
AP	0.954	-0.205	-0.161
AN	0.858	0.383	0.281
pH	-0.675	-0.478	0.408
EC	-0.660	0.644	-0.079
SWC	0.919	-0.305	-0.085
MBC	0.837	-0.389	-0.090
MBP	0.582	0.354	-0.636
MBN	0.934	0.027	-0.138
Eigenvalue	6.663	2.150	1.198
Contribution rate/%	60.572	19.543	10.890
Cumulative contribution rate/%	60.572	80.115	91.004

**Figure 5:**
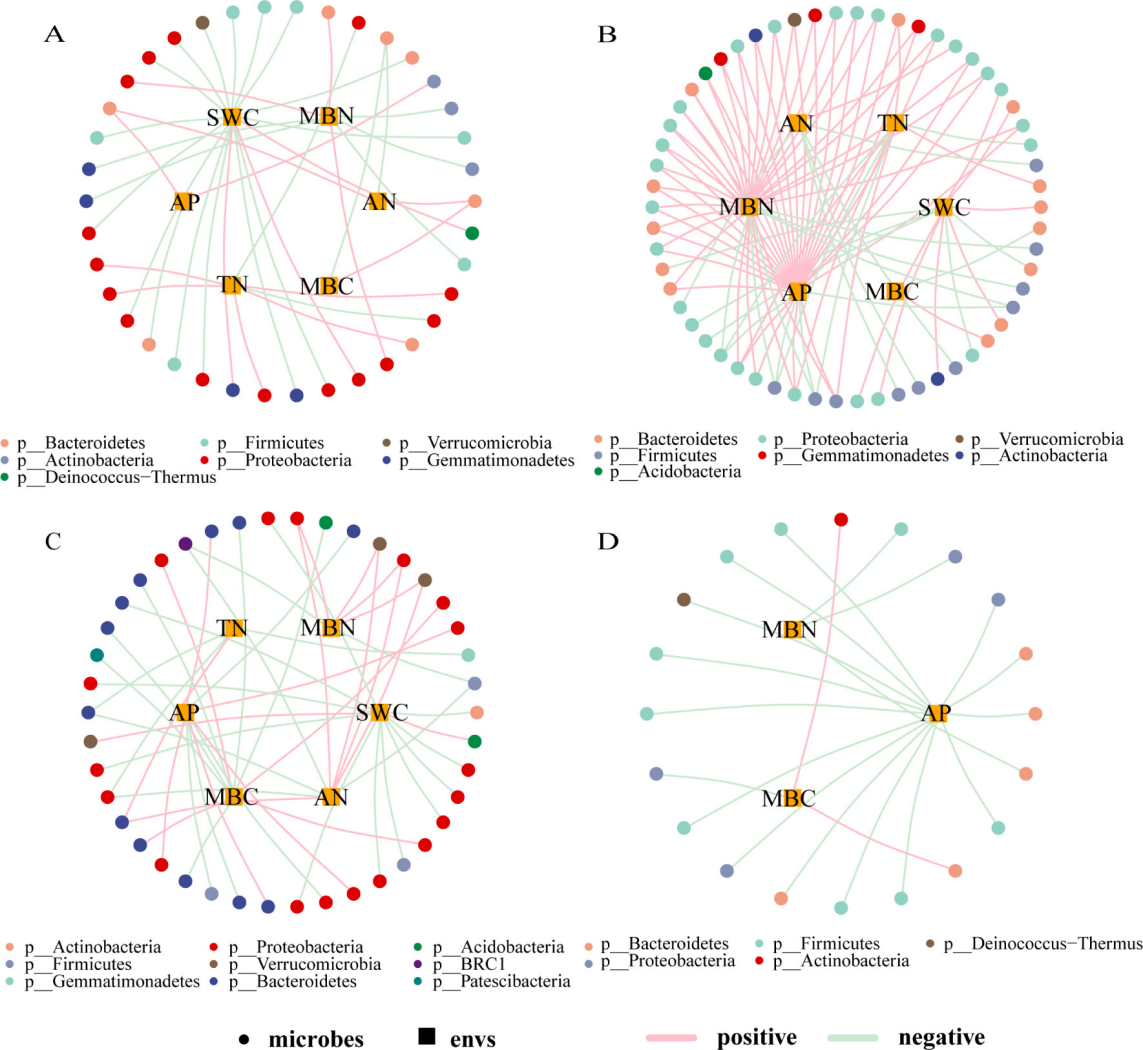
Correlation network between soil microbial species and environmental factors in different types of shelterbelts forests **(A)**: PP; **(B)**: EP; **(C)**: P; **(D)**: S. As shown in the legend, the circular nodes are species (outer circle), the square nodes are environmental factors (inner circle), the circular nodes of different colors represent genera at different gate levels, and the line colors represent correlations, with pink being positive and light green being negative.

Based on the correlation analysis between environmental factors (loadings absolute value ≥ 0.75) and microbial taxa (default parameters, genus level, Pearson correlation, correlation threshold 0.3, correlation p-value threshold 0.05, node number 80, edge number 100), a correlation network diagram was constructed, as shown in **Figure 5**. The complexity of the correlation network in EP was the highest among the four types of farmland shelterbelts, with most bacterial genera showing a positive correlation with environmental factors, and MBN and AP having a significant positive promoting effect on various bacterial genera. The bacterial genera in PP were mostly negatively correlated with environmental factors, with SWC and MBN being the main influencing factors. The relationship differences between bacterial genera and environmental factors in P were not significant, only MBC and SWC had a significant effect on various bacterial genera. The bacterial genera in S were only correlated with AP, MBN, and MBC, and most of them were negatively correlated, with AP having a more significant impact on various bacterial genera. In all four types of farmland shelterbelts, Proteobacteria were significantly influenced by environmental factors, followed by Bacteroidetes and Firmicutes.

## 4 Discussion

### 4.1 Different types of farmland shelterbelts on soil environment

Constructing farmland shelterbelts can effectively improve environmental factors within the shelterbelt system, optimize farmland soil conditions, protect vegetation cover, finally enhance the ecological benefits of farmland (Shi et al., 2016). In this study, significant differences were found in soil properties among different types of farmland shelterbelts. The pH value of S was significantly higher than the other three types farmland shelterbelts. The differences in soil pH among different tree species of farmland shelterbelts are consistent with the results of Iovieno et al. (2010), who emphasized the influence of dominant tree species on soil pH in forest stands on the same substrate. The soil SOC, TP, TN, AP, AN, SWC, MBC, MBN, and MBP contents in the P were the highest (**Table 1**), indicating excellent nutrient condition in the P soil. Then was S, with the AP, AN, MBC, and MBN contents ranking behind, suggesting that the soil nutrient element contents were also at a higher level. The soil nutrient element contents in the PP and EP two composite types of shelterbelt soil was relatively low, indicating pure forest farmland shelterbelts had better nutrient status. Given the similar growth substrates of the studied farmland shelterbelts, the differences in soil nutrient contents are likely caused by different tree species and their litter. Vegetation can adjust the microclimate through shading, frost prevention, and soil moisture regulation. Additionally, the accumulation and decomposition of plant litter, as well as the components of root exudates, including carbohydrates, amino acids, low molecular weight aliphatics and aromatics, fatty acids, enzymes, and hormones, directly or indirectly affect microbial communities, thus affecting soil nutrient contents (Prescott and Grayston, 2013). Liu and Wang (2021) compared the soil nutrients of artificial forests of *Picea crassifolia* Kom., *Larix gmelinii* var. *principis-rupprechtii* (Mayr) Pilg., *Populus cathayana* Rehder, *Betula platyphylla* Sukaczev, the results also showed significant differences in soil nutrients among different artificial forests, with the soil nutrient content of *Populus cathayana* Rehder forests being the highest. This may be because both *Populus alba* var. *pyramidalis* Bunge and *Populus cathayana* Rehder belong to broad-leaved forests, and their abundant litter results in a high input of organic matter to the soil, which is consistent with the results of previous studies (Wang et al., 2008; Shi and Cui, 2010; Lou et al., 2021). Soil humus is a substance formed by organic matter, such as plant residues, microorganisms, and other biological residues. Its content and quality have important effects on soil nutrient cycling and stability. In this study, the P soil has the lowest electrical conductivity, indicating the lowest salt content. As salinity increases, the diversity of bacterial and archaeal communities in desert soils gradually decreases (Zhang K. P. et al., 2019). The MBC, MBN, and MBP contents in the soil of P and S were significantly higher than those in PP and EP. The activity and quantity of soil microorganisms can indirectly promote an increase soil nutrient content (Wang et al., 2015), which may lead to the differences in soil nutrient contents among different tree species. The spatial distribution of the biomass of the main roots of *Populus alba* var. *pyramidalis* Bunge tends to concentrate near soil surface (Zhao et al., 2018), where a thick layer of litter covers the ground, both of which contribute to an increase in soil nutrient content. Consequently, P soil exhibits the highest nutrient content, consistent with the results of Ren et al. (2016) and Yang et al. (2018).

### 4.2 The impact of different types of farmland shelterbelts on soil microbial communities

Studying the structure and function of soil microbial communities is of great significance for deepening our understanding in the mechanisms that farmland shelterbelt ecosystems respond to environmental changes (Grosso et al., 2018). The diversity of soil microbial communities in forest land reflects their direct connection with vegetation cover, soil characteristics, and land use types (Gunina et al., 2017). In this study, the number of OTU in composite farmland shelterbelts (PP, EP) was 785 and 824, respectively, while in single-species farmland shelterbelts (P, S), it was 801 and 937, respectively. Composite farmland shelterbelts did not show a significant advantage in microbial diversity (Song et al., 2020). This may be because soil bacterial diversity is not directly related to plant diversity (Johnson et al., 2003), but rather depends more on plant specificity (Porazinska et al., 2003; Eisenhauer et al., 2010; Gunina et al., 2017). The study found significant differences in the Shannon index among the four types of farmland shelterbelts, while the Simpson index showed no significant difference. Additionally, the ACE and Chao1 indices of P were higher than those of the other three types of farmland shelterbelts. This indicates that the richness of soil microbial communities and the relative proportions of species have undergone significant changes in different types of farmland shelterbelt ecosystems, but the dominant species are similar.

In this study, the Proteobacteria was the dominant phylum in all four types of farmland shelterbelts, which is consistent with the results of a study on artificial farmland shelterbelts in the wind and sand area of northwest Liaoning Province (Zhang, 2020). Proteobacteria have the characteristics of rapid growth and tolerance to environmental changes, including changes in pH, temperature, and strong organic degradation ability. They also form mutualistic symbiotic relationships with other microorganisms, giving them a competitive advantage in soil, hence becoming the dominant phylum. At the phylum level, only the Verrucomicrobia, Acidobacteria, and Planctomycetes in P were significantly higher than the other three types of farmland shelterbelts. Verrucomicrobia has the ability to degrade cellulose and other complex carbohydrates (Nixon et al., 2019). Acidobacteria are often involved in the decomposition of plant exudates, insect remains, and dead branches and leaves in the soil (Liu et al., 2014; Wu et al., 2024), both of which contribute to the degradation of *Populus alba* var. *pyramidalis* Bunge litter. Planctomycetes promote nitrogen biogeochemical cycling (Wei et al., 2020), which positively affects nitrogen cycling and nutrient accumulation in P soils. All three benefits to the effective decomposition of litter, nutrients release and the maintenance of soil nutrient balance, providing a more favorable environment for microbial growth and forming a beneficial cycle. At the genus level, only the genus *Palleronia* in EP was significantly higher than in the other three types of farmland shelterbelts. It belongs to the Proteobacteria and exhibits good tolerance to a wide range of salinities, showing excellent adaptability (Martínez-Checa et al., 2005). The lower SWC and higher EC in EP soils may be the reasons for this result.

### 4.3 Influence factors of soil bacterial community characteristics in different types of farmland shelterbelts

Resource availability and habitat quality have significant impacts on bacterial community diversity (Yergeau et al., 2015; Wang et al., 2016). In this study, the relationship between soil microbial species and environmental factors showed significant differences among four types of farmland shelterbelts. P soils had higher levels of TN, AP, AN, SWC, MBC, and MBN, S soils had higher levels of AP, MBC, and MBN, and PP soils had higher levels of TN, AP, AN, SWC, and MBC (**Table 2**). Better soil conditions will weaken the environmental filtration (Zhou et al., 2014), reducing the dependence of species on the environment. Therefore, the correlations between microbial species and environmental factors in P, S, and PP soils were relatively simple. In contrast, more correlations were observed in the microbial species-environmental factor network in EP soil, which mainly involved SWC, MBN, and AP. Compared to other types, EP soil had lower levels of SWC, MBN, and AP, which could reduce microbial growth, maintenance, and survival rates (Marschner et al., 2001). Therefore, the microbial species in EP soil became more dependent on environmental factors, exhibiting a more complex correlation. This suggests that changes in soil bacterial communities are constrained by forest environment conditions (Gunina et al., 2017), and resource and habitat quality factors play important driving roles in regulating bacterial community diversity.

## 5 Conclusion

In this study, we comprehensively investigated the impact of different types of farmland shelterbelts on soil, and found that different tree species not only caused changes in soil properties but also influenced soil bacterial communities. In the four different types of farmland shelterbelts, the P soil nutrient content and bacterial diversity were the highest. At the same time, we observed that soil bacterial communities were regulated by soil properties. Soil TN, AP, AN, SWC, MBC, and MBN had a notable impact on bacterial community structure. Significant differences in Verrucomicrobia, Acidobacteria, Planctomycetes, and *Palleronia* were observed among different types of farmland shelterbelts. Due to the complicated effects of tree species and soil properties on soil bacterial community changes, it is difficult to distinguish the pure effects of each factor. Therefore, we conclude that the changes in soil bacterial communities are a comprehensive reflection of soil properties and tree species effects.

## Data Availability

The datasets presented in this study can be found in online repositories. The names of the repository/repositories and accession number(s) can be found below: https://www.ncbi.nlm.nih.gov/, PRJNA1123072.
